# Editorial: Investigating the elements of plant defense mechanisms within plant immune responses against pathogens

**DOI:** 10.3389/fpls.2024.1507236

**Published:** 2024-10-25

**Authors:** Katarzyna Otulak-Kozieł, Edmund Kozieł, Józef Julian Bujarski

**Affiliations:** ^1^ Department of Botany, Institute of Biology, Warsaw University of Life Sciences-SGGW, Warsaw, Poland; ^2^ Department of Biological Sciences, Northern Illinois University, DeKalb, IL, United States

**Keywords:** plant structure, physiology, signaling, plant-pathogen interactions, biotechnology, climate change, plant-disease, abiotic stress

Plants are incredible organisms that support life on Earth and serve as a basic food source for the world’s population. Unlike other organisms, plants are immobile, and their growth is closely tied to their particular environment. Their immobility forces them to constantly encounter abiotic (Bashir et al., 2021) and various biotic stressors, such as herbivores, plant viruses, and pathogenic bacteria and fungi, throughout their lifespan (Ali et al.; Jan et al.; Escalante et al.; Brelanga et al.; Rymaszewski et al.; He et al.; Aci et al.; Badami et al.; Chai et al.). Due to their exposure to these biotic factors, plants have coevolved with herbivores and pathogens, developing preformed natural barriers and inducible defense mechanisms (Jones and Dangl, 2006; Underwood, 2012; Kozieł et al., 2021). Natural, or constitutive, defenses in plants are physical barriers, such as waxy epidermal cuticles or cell walls that prevent the penetration of pathogens (Maillot et al.; Escalante et al.) or deter herbivores from feeding on generative or vegetative plant organs. Inducible defense responses, on the other hand, are activated when plants detect potential pathogens, and the speed, strength, and effectiveness of this response determine the susceptibility or resistance of a plant host (Maillot et al.; He et al.; Li et al.). This inducible response is often referred to as “basal resistance” or “innate immunity” and depends on several factors, including specific receptors (Rymaszewski et al.) that recognize pathogens or pathogen-associated elements, resistance genes (*R genes*) (He et al.; Li et al.; Rai et al.), and their products, such as NB-LRRs (Anbu et al.; Jiang et al.). These responses involve ROS generation and macromolecules like salicylic acid and glutathione (Kozieł et al.), which are crucial for initiating and directing the signal transduction about the presence of a pathogen. Additionally, the response to biotic stress is often linked to the production of specific proteins, such as mitogen-activated protein kinases, or elicitors from the plant host and the pathogen alike (Chai et al.; Brelanga et al.; Jing et al.; Zhang et al.). Disease-causing plant pathogens, including viruses, bacteria, and fungi, actively modulate different elements of plant defense mechanisms. As a result, plants and pathogens are engaged in a sophisticated molecular “arms race” that has become increasingly complex due to global climate change. These conditions have created a constant need to investigate resistance mechanisms, and their components, and to explore new methods to enhance resistance (Rai et al.; Li et al.) including external treatments with compounds like β-aminobutyric acid and γ-aminobutyric acid (Badmi et al.; Jan et al.).

Mailliot et al. investigated the transcriptome analysis of *Phytophthora capsici* infection in susceptible and partially resistant peppers. The authors identified genes that redirected resources to lipid biosynthesis, allowing partially resistant plants to subsist. Ectopic expression of the RxLR effector genes CUST_2407 and CUST_16519 in pepper lines with varying resistance levels revealed host-isolate interactions that triggered either local necrotic lesions (hypersensitive response) or leaf abscission (extreme resistance), preventing pathogen spread.

Chai et al. and Badmi et al. described new factors in host reactions to infections induced by *Botrytis elliptica* and *Botrytis cinerea*, respectively. Using transcriptomic and metabolomic analyses of *B. elliptica*-resistant *Lilium* oriental hybrid “Sorbonne”, Chai et al. identified 115 differentially accumulated metabolites (DAMs) at different stages of infection. The authors confirmed that the phenylpropanoid and flavonoid pathways play a central role in plant defense. They also concluded, using transcriptome analysis and a weighted gene co-expression network analysis (WGCNA), that jasmonic acid (JA), salicylic acid (SA), brassinolide (BR), and calcium ions (Ca^2+^) are crucial for the response of “Sorbonne” to *B. elliptica* infection. Badmi et al. explored the effect of β-aminobutyric acid (BABA) treatment on *Fragaria vesca*, revealing that BABA induces systemic susceptibility in *F. vesca*. Their transcriptome analysis suggested that genes related to “response to biological stimulus”, “photosynthesis”, and “chlorophyll biosynthesis and metabolism” were involved in this induced susceptibility of BABA-treated plants. Jan et al. investigated the use of γ-aminobutyric acid (GABA) and found that GABA treatment activated antioxidant enzymes, reduced reactive oxygen species and malondialdehyde levels, and decreased the rate of damage caused by *Sogatella furcifera*. Interestingly, GABA-treated plants infested with *S. furcifera* also exhibited increased phenylalanine ammonia-lyase and pathogenesis-related (PR) gene expression levels, and GABA-induced abscisic acid (ABA) accumulation, stomatal closure, and reduced water conductance in leaf vessels during stress caused by *Sogatella furcifera*. Furthermore, GABA induced the expression of JA biosynthesis genes (*LOX, AOS, AOC*, and *OPR*) and melatonin biosynthesis-related genes (*TDC, T5H, ASMT*, and *SNAT*).

The data presented by Zhang et al. showed the direct role of the LysM protein BdLM1 of *Botryosphaeria dothidea* in full virulence and the inhibition of plant immunity by binding chitin and protecting hyphae from hydrolysis. Jing et al. postulated that plasma membrane (PM) dynamics play a role in defense against pathogens and explained the signaling pathway of plant elicitor peptides (Peps) and their effect on PM protein internalization. The authors demonstrated that Pep1 stimulates the endocytosis of PM-localized proteins through clathrin-mediated endocytosis (CME). CLC2 and CLC3, two light chains of clathrin, are vital for Pep1-induced PIN2-GFP and BRI1-GFP. The internalized PIN2 and BRI1 are subsequently transported to the vacuole via the trans-Golgi network/early endosome (TGN/EE) and pre-vacuolar compartment (PVC) pathways. Moreover, Jing et al. showed that salicylic acid (SA) negatively regulates the effect of Pep1 on PM endocytosis. Furthermore, Rymaszewski et al. revealed that HopQ1, a type three effector from *Pseudomonas syringae*, upon phosphorylation, co-opts plant 14-3-3 proteins to control its stability and subcellular localization, affecting the nuclear import rate of the *Pseudomonas syringae* effector in *Nicotiana benthamiana* cells. On the other hand, the analyses of Jiang et al. on *Pseudomonas syringae* pv. *actinidiae* (PSA) focused on the role of overexpression of miRNA482 family, miRNA-215-3p, and miRNA-29-3p in increasing kiwifruit’s sensitivity to PSA via regulation of NBS-LRR target genes.

He et al. analyzed the further role of the *R* executor genes, *Xa7, Xa10*, *Xa23*, and *Xa27* in infection caused by *Xanthomonas oryzae* pv. *Oryzae* (*Xoo*). The authors confirmed that transcription activator-like effector (TALE) AvrXa7 in *Xoo* strains could bind directly to the effector-binding element (EBE) in the promoter of the *Xa7* gene. Moreover, the executor *R* genes (*Xa7*, *Xa10*, *Xa23*, and *Xa27*) driven by the promoter of the *Xa7* gene trigger the hypersensitive response (HR) in tobacco leaves. Berlanga et al. confirmed the extensive role of mitogen-activated protein kinase phosphatase 1 in controlling broad-spectrum antibacterial and antifungal resistance in *Arabidopsis thaliana* through diverse mechanisms of immune activation. Meanwhile, the analyses of comparative transcriptome profiling and co-expression network analysis performed by Aci et al. revealed the key genes associated with pear petal defense responses against *Monilinia laxa* infection in Sissy (relatively tolerant cultivar) and Kristalli (highly susceptible cultivar). Li et al. characterized the interaction between eggplant and *Verticillium dahliae*, in particular the highly resistant cultivar LC-2 with higher levels of polyphenol oxidase, superoxide dismutase, peroxidase, phenylalanine ammonia lyase, β-1,3 glucanase, or chitinase. Meanwhile, RNA sequencing performed by Li et al. revealed differentially expressed genes (DEGs), a significant portion of which were implicated in disease resistance and growth. These processes encompassed defense responses, cell wall biogenesis, developmental processes, and the biosynthesis of spermidine, cinnamic acid, or cutin. Rai et al. characterized susceptible and resistant cultivars of *Brassica juncea* against *Albugo candida* with special effort on antioxidant enzymes and non-enzymatic ROS scavenging compounds. The authors emphasis PR2 as the best possible gene for defense against *A. candida* followed by PR1, while PR3 and PR12 showed a positive correlation with the disease resistance, which may be due to the jasmonate pathway acting as a complement to the salicylic acid pathway. On the other hand, Li et al. elucidated the pathogenesis of powdery mildew in various susceptible varieties of *Ribes nigrum* L, through the observation of postinfection physiological changes, and molecular mechanisms related to powdery mildew. Moreover, the authors demonstrated that flavanone 3-hydroxylase (F3H) and dihydroflavonol reductase (DFR) positively regulate powdery mildew resistance, while anthocyanin reductase (ANR) and polygalacturonase (PG) play a role as negatively regulated factors.

Taken together, these studies provide new and interesting insights into plant-microbe interactions and their implications for understanding how pathogens change adaptive mechanisms to infection or how plants develop diverse resistance. Therefore, there is a strong need for further research in this area which will provide scientific support to improve disease prevention and control in plants.
